# Dynamic players and intricate interactions: An integrated investigation of the Mla lipid transport system

**DOI:** 10.1016/j.jbc.2023.105146

**Published:** 2023-08-09

**Authors:** Dixon Ng, Trevor F. Moraes

**Affiliations:** Department of Biochemistry, University of Toronto, Toronto, Ontario, Canada

## Abstract

The Maintenance of outer membrane (OM) Lipid Asymmetry system mediates retrograde phospholipid transport from the OM to the inner membrane (IM) in Gram-negative bacteria. However, the interactions between the various subunits of the IM and OM complexes are not well understood. In a recent study in 2023 by MacRae *et al*. in the *Journal of Biological Chemistry*, the authors examine components in the Maintenance of OM Lipid Asymmetry complex, define the interaction interfaces between members of the pathway, and propose a molecular model of the lipid transfer process from the OM to the IM that will help elucidate intricacies of lipid transport.

Lipid asymmetry of the Gram-negative outer membrane (OM) is a critical feature that provides protection against environmental stresses, including antibiotics and host immune responses (1, 2, 3). The Mla system is comprised of three components: an MlaFEDB ABC transporter complex in the inner membrane (IM), a periplasmic chaperone MlaC, and MlaA, which is complexed with porin protein OmpF/C in the OM (4, 5) (Fig. 1). The MlaA–OmpF/C complex in the OM recognizes mislocalized phospholipids (PLs) and prepares them for shuttling back to the IM (6). To coordinate this transport, MlaA recruits and binds MlaC, whose role is to transport the PLs across the periplasm to the MlaFEDB complex in the IM. The IM complex accepts the PLs from MlaC and powers the incorporation of the lipids into the IM by ATP hydrolysis (7, 8).

**Figure 1:**
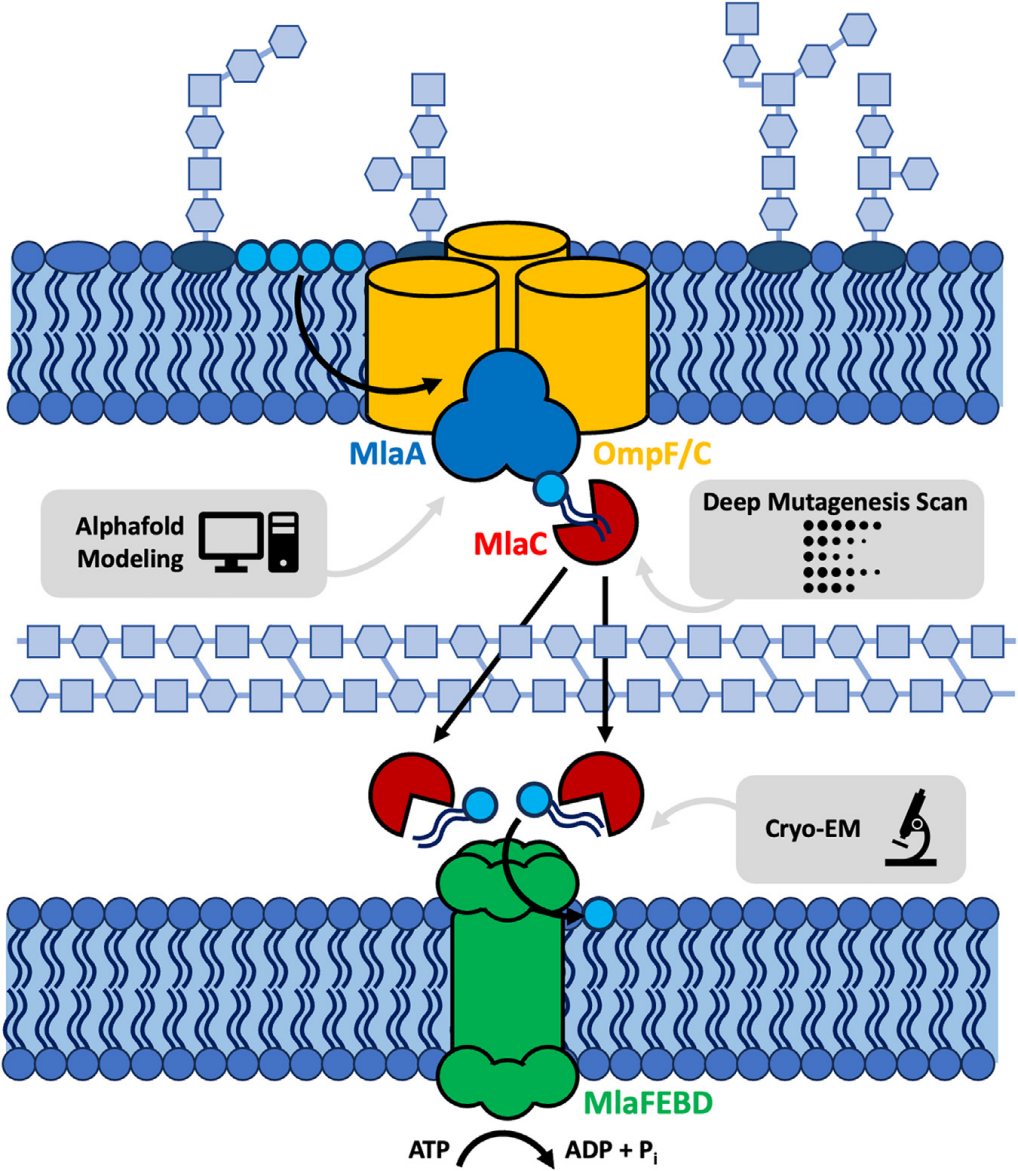
**An integrative approach using AlphaFold2 modeling, deep scanning mutagenesis, growth assays, and state-of-the-art structural biology techniques reveals the dynamic players of the** Mla **system and elucidates their interactions and lipid transport mechanism.** The study focuses on investigating the interactions and interfaces between the periplasmic lipid shuttle MlaC and the outer and inner membrane components.

Understanding the mechanisms that underlie the asymmetric nature of the OM provides valuable insights into the maintenance process. In their recent study, MacRae *et al.* (9) address a significant knowledge gap in our understanding of the protein–protein interactions between components of the Mla lipid transport system. In lieu of atomic-resolution structural data of the Mla complex that has been difficult to achieve because of the heterogeneous nature of the complexes and their transient interactions, the authors took an integrative approach that combines clever high-throughput biochemical assays, low-resolution structural data, and predictive structural modeling to advance our understanding of how the Mla proteins interact during the lipid transfer process.

To unravel the mechanism of the lipid transport complex, MacRae *et al*. highlighted all the critical residues involved in complex formation and PL transport through an extensive deep mutagenesis scan consisting of 4000+ MlaC variants. Critical residues were mapped onto structural models created by Alphafold2 to validate clusters on MlaC and to elucidate the functional interactions between MlaA, MlaC, and the MlaFEDB complex. Although the cryo-EM density maps in this study had limited resolution, it became evident that up to two MlaC molecules can bind simultaneously onto the IM-anchored MlaD during the handoff process.

The multidisciplinary approach in this study showcases the potential of integrative methodologies in studying complex biological systems and exemplifies how we can probe intricate protein–protein interactions even in the absence of high-resolution structural data. The current artificial intelligence revolution has brought about remarkable advances in structural modeling, as demonstrated by AlphaFold2's unprecedented levels of accuracy in predicting protein structures and interaction interfaces (10).

Transient interactions between cargo carriers like MlaC and membrane transporters or enzymes are challenging to define; furthermore, our predictions show that many proteins including membrane proteins contain intrinsically disordered regions that are important in dynamic protein–protein interactions (11). In the Mla system, where MlaC is shown to hand off the lipid to the MlaFEDB complex, the C-terminal helices and tail region of MlaD is predicted to be disordered. New algorithms and tools (12), that aid in modeling these regions (13) will play important roles when paired with experimental validations, as exemplified in this study, and will help bridge our understanding of transient and dynamics processes within the cell, including lipid transport.

Despite the progress made in understanding the Mla system and lipid recycling in Gram-negative bacteria, there are several important aspects that remain to be investigated. First, it is unclear how the OM complex selects lipids for recycling and whether the process is stochastic or directed. Second, the mechanism by which MlaC traverses the peptidoglycan-rich periplasm is still unknown. In addition, the process of lipid removal from MlaC and the role of ATP hydrolysis in lipid release and adsorption into the IM need further atomic resolution for mechanistic elucidation. The authors’ identification of clusters of surface-exposed residues on MlaC that are not part of the two interfaces (lipid binding or Mla system binding) hints at the presence of other interacting partners, raising the question of their involvement in the lipid transport process. The recent discovery of MlaZ/Y system provides information about an additional mechanism to maintain OM asymmetry by removing and degrading mislocalized OM lipids through the lipase function of the OM lipoprotein MlaY in concert with the OM protein MlaZ (14). This further emphasizes how bacteria value the importance of maintaining lipid asymmetry in OM.

Through a better understanding of the Mla pathway and OM lipid recycling, we gain understanding of bacterial membrane dynamics and expand our knowledge of how these molecular machineries function together to maintain OM lipid asymmetry. These insights are crucial for the development of new therapeutics and the enhancement of antimicrobial compound efficacy by facilitating better penetration through the OM.

## Conflict of interest

The authors declare that they have no conflicts of interest with the contents of this article.
